# Multidisciplinary Treatments, Patient Characteristics, Context of Care, and Adverse Incidents in Older, Hospitalized Adults

**DOI:** 10.1155/2012/350830

**Published:** 2012-03-11

**Authors:** Leah L. Shever, Marita G. Titler

**Affiliations:** ^1^Nursing Research, Quality, and Innovation, University of Michigan Health System, 300 North Ingalls, Room NI 5A07, Ann Arbor, MI 48109-5446, USA; ^2^University of Michigan School of Nursing and University of Michigan Health System, 400 North Ingalls, Suite 4170, Ann Arbor, MI 48109-5482, USA

## Abstract

The purpose of this study was to examine factors that contribute to adverse incidents by creating a model that included patient characteristics, clinical conditions, nursing unit context of care variables, medical treatments, pharmaceutical treatments, and nursing treatments. Data were abstracted from electronic, administrative, and clinical data repositories. The sample included older adults hospitalized during a four-year period at one, academic medical facility in the Midwestern United States who were at risk for falling. Relational databases were built and a multistep, statistical model building analytic process was used. Total registered nurse (RN) hours per patient day (HPPD) and HPPDs dropping below the nursing unit average were significant explanatory variables for experiencing an adverse incident. The number of medical and pharmaceutical treatments that a patient received during hospitalization as well as many specific nursing treatments (e.g., restraint use, neurological monitoring) were also contributors to experiencing an adverse incident.

## 1. Background

The Institute of Medicine (IOM) report *To Err is Human *[1] revealed the number and significance of adverse events and errors that occur during hospitalization. The report was a call to action to transform healthcare systems to ensure patient safety and higher quality care. In one step toward healthcare transformation, the Centers for Medicare and Medicaid (CMS) no longer reimburses institutions for the care, or treatment, associated with certain hospital-acquired conditions [2].

 Understanding what factors contribute to adverse incidents during hospitalization is essential to developing effective counter measures. In order to improve factors that are modifiable within a hospital structure or with healthcare delivery, it is important to first have an understanding of what is broken. There are a number of potential contributing factors that need to be considered such as the patient's condition, the care the patient receives, and the environment in which they receive care [3, 4]. 

Battles and Lilford [3] provide a conceptual model for patient safety that includes antecedent conditions, which would include the patient's comorbid conditions, the primary reason the patient was admitted to the hospital, and characteristics the patient possessed before entering the hospital. Their model also includes the structure, or environment, in which the patient receives care such as the hospital, or nursing unit. Also acting within the structure are the processes of care (the interventions or treatments) delivered by the multidisciplinary team caring for the patient in the hospital. None of these components exist in isolation, which is why it is important to examine all of these factors and how they interact [3].

## 2. Purpose

**Figure 1:**
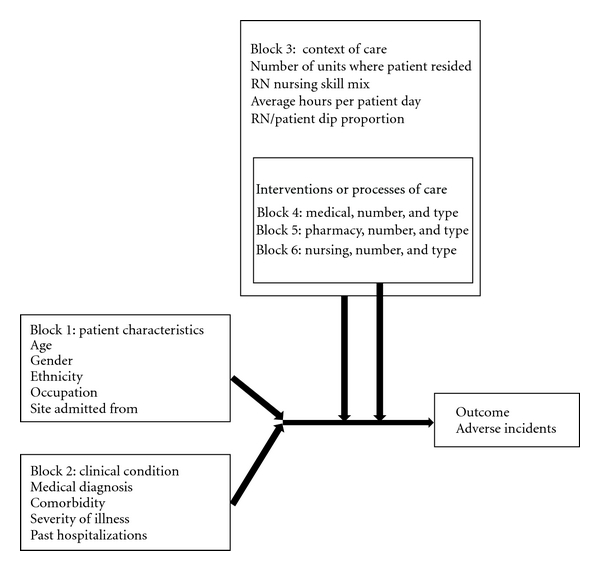
Model for predicting adverse incidents in the hospital.

The purpose of this study was to examine factors that contribute to adverse incidents that occur during hospitalization by creating a model that included patient characteristics, clinical conditions, nursing unit context of care variables, medical treatments, pharmaceutical treatments, and nursing treatments. The research question addressed in this study is: what patient characteristics, clinical conditions, context of nursing care variables (e.g., nursing hours per patient day, RN skill mix, number of units resided on during hospitalization), and treatments (medical, pharmaceutical, and nursing treatments) explain the occurrence of adverse incidents for hospitalized, older adults at risk for falling? A model that has been used successfully to guide multidisciplinary effectiveness research in the hospital setting can be seen in Figure 1 [5, 6].

## 3. Methods

Data for this exploratory study came from a large, health service effectiveness grant [7] and was approved by the institution's Human Subjects review board. Data from a four-year period (July 1, 1998 to June 31, 2002) were extracted for the primary study from one large Midwestern academic medical center. Data sources came from nine electronic data repositories, including the nursing information system that used the Nursing Interventions Classification (NIC) [8] to electronically document nursing care delivered. Detail of the nine electronic repositories and methods to assure validity and reliability are discussed elsewhere [5]. Extracted data were stored in a structured query language (SQL) server and relational databases were built using a unique subject number.

### 3.1. Sample

The inclusion criteria were hospitalizations to one Midwestern tertiary care hospital over a four-year period, patients 60 years of age or older upon admission, and at risk of falling. Patients were determined to be at risk of falling based on a fall risk assessment [6] that was completed upon admission or when the patient received the nursing intervention of Fall Prevention as recorded in the electronic documentation system. Patients at risk for falling were selected with the rationale that they would be at risk for experiencing one adverse incident (i.e., falling), and therefore interventions would be initiated to prevent the adverse incident. In addition, the hospitalizations were selected as the unit of analysis rather than individual patients and a variable was included to control for patients who had more than one hospitalization.

### 3.2. Study Variables

Conceptual and operational definitions for the independent variables included in the explanatory model are displayed in Table 1 and organized by the conceptual model seen in Figure 1 (patient characteristics, clinical conditions, context of care, and treatments). When appropriate, the source used to guide coding of variables is provided; for example, pharmaceutical treatments, or medications, were coded using the American Hospital Formulary Service (AHFS) codes [9]. 

The dependent variable for this analysis was the first occurrence of an adverse incident during an episode of hospitalization. Adverse incidents were defined as any undesired circumstance that lead to, or could have led to, personal harm. Adverse incidents were collected by the internal incident reporting system at the institution. Adverse incidents included falls, medication errors, procedure-related events (e.g., wrong patient, wrong procedure or test), equipment-related events (e.g., equipment malfunction, unplanned removal, improper set-up), and new conditions (e.g., skin breakdown). 

## 4. Analytic Procedures

Due to the large number of study variables, a four-step model building process using logistic regression was used to answer the research question.

### 4.1. Step One

Each independent variable included in the analysis was tested independently using a bivariate analysis and a Score Statistic to determine the association with occurrence of an adverse incident. In this bivariate analysis, no other variables were statistically controlled for. Variables with *P* values ≤0.15 were retained for step two. A *P* value ≤0.15 was used as the criterion to guard against eliminating variables too soon in this exploratory analysis.

### 4.2. Step Two

The variables retained in step one (*P* values ≤0.15) were then analyzed within their respective conceptual variable blocks (i.e., patient characteristics, clinical conditions, context of care, medical treatments, pharmaceutical treatments, and nursing treatments) using logistic regression. A backward elimination process was used, indicating that the variable with the largest *P* value was eliminated and the analysis was rerun on the remaining variables within the block. This procedure was repeated until all variables within the block had a *P* value ≤0.15. A *P* value of ≤0.15 during step two was chosen to guard against eliminating variables too soon because they might yet prove to have a statistically significant effect when combined with variables from other conceptual blocks.

### 4.3. Step Three

A model integrating all of the conceptual variable blocks was built in a progressive fashion using the variables that were retained in step two. The significant variables were added to the model by their respective blocks. Starting with the significant variables in block one (patient characteristics) and block 2 (clinical conditions), a model was built using the backward elimination process described in step two until the only variables remaining in the model were those with a *P* value ≤0.15. The significant variables from block three (context of care) were then added to what remained of blocks one (patient characteristics) and two (clinical conditions) in the model. Once again, a backward elimination process was performed until the only variables remaining in the model were those with values ≤ 0.15. This process of adding blocks and using the backward elimination continued until the last block (nursing treatments) was added. At this point, when the significant variables from the final block were added and backward elimination was performed, the criterion for significance was decreased to a *P* value ≤0.05. This resulted in a final model containing only those variables with a *P* value ≤0.05. In step three, variables with a *P* value ≤0.05 in the logistic regression indicated that variables were significantly related to the dependent variable (occurrence of an adverse incident) after controlling for the other variables in the model.

### 4.4. Step Four

Covariates used for risk adjustment included age, severity of illness, and number of hospitalizations during the study period (see Table 2). Step four added these covariates used for risk adjustment (severity of illness, age, and more than one hospitalization during the study period) to the model to those that were significant in step three. Categorical variables with more than two categories were analyzed by comparing each level to a reference category. For example, severity of illness (four levels from minor to severe) was analyzed by comparing each of the three upper level categories to the lowest level of severity of illness (i.e., minor).

## 5. Results

There were 10,157 hospitalizations included in this analysis, comprised of 7,851 unique patients. The mean age was 73.7 years; most were retired (74.4%), Caucasian (93.5%), female (52.6%), and admitted from home (64.4%). This patient group, defined primarily by receiving the nursing treatment Fall Prevention, was medically diverse. The most common primary medical diagnoses were diseases of the circulatory system (28.5%), neoplasms (13.8%), and injury, including fractures, or poisoning (11.5%).

There were 1,568 hospitalizations that experienced at least one adverse incident in this sample. The most commonly experienced adverse incident for this patient group included medication errors (37%), falls (27%), and equipment-related events (14%).

Results of the model building process are illustrated in Table 2 by variable blocks. The bivariate correlations completed in step one are not included in Table 2 due to space constraints but are available from the authors upon request. The second column in Table 2 illustrates variables retained from step one that were analyzed within blocks with *P* values ≤0.15 (step two of model building) and thus retained for step three. The third column includes *P* values from the third part of the modeling building process, prior to adding covariates used for risk adjustment to the final model (step four). The final model is illustrated in Table 3.

Five patient characteristics entered step one of the model building process but none were significant beyond step two. *Age*, although not significant in any of the three model building steps, was entered in the final model for risk adjustment [17]. *Age* was not significant in the final model (see Table 3).

Nine primary medical diagnoses were retained from step two, four were retained from step three, and three were retained (*P* ≤ 0.05) in the final model (see Tables 2 and 3). As the results in Table 3 indicate, *other nervous system disorders, other primary cancer *and* senility and organic mental disorders* were all significant (*P* ≤ 0.05) in the final model. *Other nervous system disorders* was the only primary medical diagnosis of the three inversely associated with experiencing an adverse incident (O.R. = 0.43), indicating that hospitalizations with this medical diagnosis were less likely to suffer an adverse incident compared to hospitalizations that did not have this condition. O*ther primary cancer* and *senility and organic mental disorders* were both positively associated with experiencing an adverse incident with odds ratios of 1.94 and 1.57, respectively.

Severity of illness, although not significant in step three, was entered into the final model for risk adjustment [17]. *Severe* and *major* severity of illness categories were significantly (*P* ≤ 0.05) and positively associated with experiencing an adverse incident compared to the lowest severity of illness category (i.e., mild) (see Table 3). 

Seven comorbid conditions were retained from step two for inclusion in step three but none were significant and thus were not retained for inclusion in the final model. Past hospitalizations during the study period were significant in step two but not in step three (see Table 2). However, this variable was entered into the final model to adjust for patients that had experienced more than one hospitalization during the study period. In the final model (Table 3) past hospitalizations were not significant. 

Four context of care variables, the *number of units the patient resided on during hospitalization*, the *dip proportion (falling below the unit's average staffing)*, *skill mix*, and the *average Caregiver Patient Ratio (CGPR)* [14], were significant in step two (see Table 2) but only two variables, the *dip proportion* and *average CGPR,* were significant in step three and retained for the final model (see Table 2). Both were significant in the final model (step four) as illustrated in Table 3. The *average CGPR* (RN hours per patient day (HPPDs)) was categorized as quartiles to enable comparison and interpretation for this nonlinear variable. The two highest *average CGPR* quartiles (9.5 RN HPPDs and 6.6 RN HPPDs) were significantly (*P* ≤ 0.05) and inversely associated with experiencing an adverse incident, indicating that when compared to the lowest quartile of staffing (4.1 RN HPPDs), the odds of experiencing an adverse incident decreased in the highest two quartiles of nursing hours per patient day. The odds of experiencing an adverse incident for hospitalizations with the highest *average CGPR* quartile (9.5 RN HPPDs) were 0.76 of the odds for hospitalizations that experienced the lowest *average CGPR* quartile (4.1 RN HPPDs). The odds of experiencing an adverse incident for hospitalizations with the second highest *average CGPR* (6.6 RN HPPDs) were 0.62 of the odds for hospitalizations in the lowest *CGPR average* quartile.

The CGPR dip proportion was significantly   (*P*  =  0.011) and positively associated with experiencing an adverse incident. The results shown in Table 3 are in terms of 0.2 increments of change and indicate that for each 20% fall in staffing below the average, the odds of experiencing an adverse incident increase by 15%  (O.R. = 1.15).

The number of medical treatments received during hospitalization and 20 types of medical treatment were significant in step two (see Table 2) and were therefore included in step three. In step three of the analysis, the number of medical treatments received during hospitalization and one medical treatment type, *physical therapy*, were significant (*P* ≤ 0.05) and retained for the final model. Both were positively associated with experiencing an adverse incident (see Tables 2 and 3). The results indicate that for each additional medical treatment received during hospitalization, the odds of experiencing an adverse incident increased by approximately 3%  (O.R.  = 1.03). Hospitalizations that received the medical treatment *physical therapy* were 52%  (O.R.  = 1.52) more likely to experience an adverse incident than hospitalizations that did not receive this medical treatment.

The number of unique medications received during hospitalization and 27 specific pharmaceutical treatments (i.e., medications types) were significant in step two of the analysis (*P* ≤ 0.15) and thus retained for step three. The number of unique medication types and four types of medications were significant in step three (see Table 2) and all were significant in the final model (see Table 3). The number of unique medications was positively associated (*P* < 0.001) with experiencing an adverse incident (O.R. =  1.04). Receipt of *succinimides, caloric agents, *and *EENT anti-infectives *during hospitalization increased the odds of an adverse incident. *Ammonia detoxicants *were inversely associated (*P* = 0.021) with experiencing an adverse incident (O.R. = 0.46). 

In step two of the analysis, the number of unique nursing treatments received during hospitalization was not significant but 38 types of nursing treatments were significant (*P* ≤ 0.15) and entered into step three (see Table 2). Eleven were significant at step three and ten were significant in the final model (see Tables 2 and 3). *Surgical preparation* was not significant in the final model. The nursing treatment *pressure ulcer care,* received by 91.5% of the sample, was divided into thirds based on the average number of times per day it was delivered (see Table 1). The results for the three categories of use are interpreted in comparison to hospitalizations that did not receive the nursing treatment. The middle and low use categories of *pressure ulcer care *were significantly (*P* ≤ 0.05) and positively associated with experiencing an adverse incident, indicating that hospitalizations that received *pressure ulcer care *a little less than once every other day (use rate = 0.41) or once every four days (use rate = 0.25) were more likely to experience an adverse incident than hospitalizations that did not receive *pressure ulcer care.* A similar pattern emerged with the nursing treatment of *specimen management. *The medium (use rate = 0.34) and low (use rate = 0.10) categories were significantly   (*P* ≤ 0.05) and positively associated with experiencing an adverse incident (see Table 3). 

Both *health screening *and *neurologic monitoring* had low use categories that were significantly   (*P* ≤ 0.05) and positively correlated with experiencing an adverse incident. The results indicate that hospitalizations that received the low use of these two nursing treatments were more likely to experience an adverse incident than hospitalizations that did not receive the associated nursing treatment (see Table 3). 

The medium use category of *blood products administration* (use rate = 0.89) was significantly (*P* ≤ 0.05) and positively (O.R. = 1.49) associated with experiencing an adverse incident. Hospitalizations that received *Blood Products Administration *a little less than once a day were almost 50% more likely to experience an adverse incident than hospitalizations that did not receive *blood products administration.*

All three categories of use for the nursing treatment *restraint* were significantly (*P* < 0.01) and positively associated with experiencing an adverse incident (see Table 3). The high use category had an average delivery of 16.47 times a day and hospitalizations that received high use of *restraint* had more than double the odds (O.R. = 2.16) of experiencing an adverse incident compared to hospitalizations that did not receive this nursing treatment. Hospitalizations that received *restraint* approximately four and a half times a day (medium use category) had almost double the odds (O.R. = 1.86) of experiencing an adverse incident compared to hospitalizations that did not receive *restraint*. The lowest category of use was delivered an average a little more than once a day and increased the likelihood of experiencing an adverse incident by 58%  (O.R. = 1.58) compared to no use.

The remaining significant nursing treatments were delivered to less than 5% of the sample and were therefore operationalized as dichotomous variables so that hospitalizations that received the nursing treatment at least once are compared to hospitalizations that did not receive the treatment (see Table 1 for definition). *Active listening* received at least once by 4.8% of the sample was significantly (*P* < 0.001) and positively (O.R. = 1.63) associated with experiencing an adverse incident. 

*Mood management *was received by only 2.5% of the sample but was delivered an average of 3.1 times per day when it was delivered. Hospitalizations that received *mood management *almost doubled their odds (O.R. = 1.84) of experiencing an adverse incident compared to hospitalizations that did not receive *mood management*. 

*Cast care maintenance* was another nursing treatment that was delivered frequently (more than five times a day on average) when hospitalizations required it. Receiving this nursing treatment doubled the odds (O.R. = 2.00) of experiencing an adverse incident compared to hospitalizations that did not receive this nursing treatment.

Slightly more than one percent of the sample received the nursing treatment *music therapy*. The average use rate for hospitalizations that received this treatment was slightly more than once every ten days (use rate = 0.21). The odds of experiencing an adverse incident were double (O.R.  =  2.03) for hospitalizations that received this nursing treatment compared to hospitalizations that did not receive *music therapy *(see Table 3). 

## 6. Discussion

None of the patient characteristics were significant, indicating that patient characteristics were not explanatory variables of adverse incidents, given the other variables that entered the model. Also nonsignificant were two clinical conditions: number of past hospitalizations during the study period and comorbid medical conditions. This indicates that after controlling for other variables in the model, patient characteristics of this sample of older adults were not significant for experiencing an adverse incident during hospitalization. 

Three primary medical diagnoses were significant explanatory variables associated with experiencing an adverse incident. *Other nervous system disorders* were inversely associated with experiencing an adverse incident. This inverse relationship may be explained by considering the type of nursing unit these patients are typically admitted to. A primary medical diagnosis of *nervous system disorder,* which is composed of peripheral and central nervous system disorders along with more generic symptoms of a nervous system disorder [11], would likely warrant admission to a neurology unit in this academic medical setting where the nursing personnel are skilled in the care of these patients and may recognize the need for increased surveillance. This heightened surveillance for these specialized patients may decrease adverse incidents. 

*Other primary cancer *was positively associated with experiencing an adverse incident. Patients hospitalized with the primary medical diagnosis of *other primary cancer* are on high-risk medications, some that call for double-checks, and that may increase the number of medication errors that are discovered. The third primary medical diagnosis, *senility and organic mental disorders,* appears similar in nature to *other nervous system disorders* but is positively associated with experiencing an adverse incident, unlike *other nervous system disorders*. This may be because patients who have *senility and organic mental disorders* are less capable of using safety equipment in their environment like call lights and hand rails and are more likely to be dispersed among a variety of general medical or surgical units. The environment and specialized nursing expertise may not be readily available to meet the unique care demands of individuals with this primary medical condition. In the final model, the top two severities of illness categories (i.e., severe and major) were significantly and positively associated with experiencing an adverse incident. This is not surprising, as patients who are sicker often have complex care issues which may place them at greater risk to experience an adverse incident. 

Related to the structure of care (context of care), the two highest categories of the *average CGPR* (RN HPPDs) were significantly and inversely associated with experiencing an adverse incident compared to the lowest quartile, indicating that when there are more nursing hours per patient day, there is a decreased likelihood of preventing an adverse incident. This is consistent with findings from previous research [18–24]. 

The *CGPR RN dip proportion *was positively associated with adverse incidents. The more the RN staffing fell below the nursing unit average, the more likely an adverse incident was to occur during that hospitalization. This finding indicates that not only is the number of nurses, or HPPDs, an important predictor of adverse incidents but so is staffing below the average on a nursing unit. This may indicate that units develop effective processes dependent upon their average staffing and when the staffing is altered, the processes are impacted. Staffing below the unit average places the patient at greater risk for having an adverse incident.

Processes of care included medical, pharmaceutical, and nursing treatments. Both the number of medical treatments and the number of unique medications received during hospitalization were positively associated with experiencing an adverse incident. As the number of procedures and medications increased so did the odds of having an adverse incident (e.g., medication error, wrong site surgery, trauma, etc.).

There was one medical treatment, *physical therapy, *and two medication types, *succinimides* and *ammonia detoxicants, *that were significantly associated with experiencing an adverse incident, which may be related to falls. The positive association between *physical therapy* and adverse incidents may be a reflection of patients with decreased functional status who are at greater risk for falling. Similarly, s*uccinimides* are anticonvulsives and are in the same AHFS class as *barbiturates* and *benzodiazepines *[9], which are positively associated with falls [25]. *Ammonia detoxicants* was the only pharmaceutical treatment in the final model inversely associated with experiencing an adverse incident (see Table 3). Patients who require *ammonia detoxicants* often have conditions associated with liver dysfunction, which makes it more difficult for them to excrete ammonia that builds up in their body. Patients that have high ammonia levels are often confused, disoriented, difficult to direct, and are at great risk for falling for these reasons.

The nursing treatments associated with adverse incidents were diverse. There was one nursing treatment, *pressure ulcer care*, that is used to treat an adverse incident (i.e., pressure ulcer). There were also a number of nursing treatments positively associated with adverse incidents where providing the treatment showed that the patient likely had greater exposure to an adverse incident than patients who did not receive the treatment. One example is the nursing treatment *specimen management *where a patient is more likely to have a mislabeled lab as an adverse incident than a patient who did not receive this treatment. The same could be true for *blood product administration *and* cast care maintenance.*

Similarly, all three categories of *restraint* were significantly and positively associated with experiencing an adverse incident. Only 8.5% of the hospitalizations in this sample received *restraint* at least once but the use rates were relatively high, especially the high use category with an average delivery of 16.47 times per day. These findings also show that use of restraints does not prevent adverse incidents (e.g., falls) and in fact may contribute to them as has been demonstrated in other research [26, 27]. 

*Active listening, mood management, *and *music therapy *may be used as complementary therapies for patients who are distressed, confused, or combative when other treatments have not worked. Hospitalizations that require these nursing treatments may be at greater risk for falling because the patient is unable to follow commands, is impulsive or unable to communicate effectively.

## 7. Limitations

This study was conducted at one academic medical center and therefore further multisite research is needed. Although the effectiveness research model used in this study includes many important, patient and multidisciplinary components, there were important aspects of care that impact patient safety such as the individual characteristics of the clinicians involved in care (e.g., experience, education) and how they interact with one another (e.g., teamwork, communication) that were not included in this study [28].

## 8. Conclusion

This study examined a number of patient conditions, structural variables, and process of care variables to better understand what factors contribute to adverse incidents during hospitalization. This is one of the first studies to show that delivered nursing treatments help explain adverse incidents in hospitalized, older adults. This study also used a multidisciplinary model that considered medical and pharmaceutical components of treatment, which are critical when providing care of the older adult in acute care. With this more robust multidisciplinary model, RN staffing was still an important explanatory variable for adverse incidents, which is congruent with findings from other research [29, 30].

## Figures and Tables

**Table 1:** Independent variable definitions.

Variable name	Variable definition and coding source	Variable type and operational definition
Patient characteristics		
Gender	The behavioral, cultural, and psychological traits typically associated with one's sex.	Categorical: M = male, F = female, D = deferred (not determined yet).
Age	Age when patient was admitted to hospital.	Continuous; measured in years.
Occupation	Activity pursued as a livelihood.	Categorical: 1 = retired, 2 = working/employed, 3 = homemaker, 4 = not retired/not employed.
Ethnicity	Race: a group of people united by certain characteristics.	Categorical: 1 = Caucasian, 2 = all others (includes the categories of African American, Hispanic, Native American/Alaskan Native; Asian/Pacific Islander, and other).
Site admitted from	The site from which the patient was admitted to the hospital.	Categorical: 1 = hospital, 2 = care facility, 3 = home/other routine admission.

Clinical conditions		
Primary medical diagnosis	The primary medical diagnoses came from the International Classification of Diseases, 9th Revision (Clinical Modification) (ICD-9-CM) codes [10] found in MRA diagnostic codes and have been classified into Clinical Classification Software (CCS) categories [11].	Dichotomous:0 = no, the diagnosis (i.e., as represented by a particular CCS category) is not the primary diagnosis, 1= yes, the diagnosis (i.e., as represented by a particular CCS category) is the primary diagnosis.
Severity of illness	A rating assigned to each hospital visit retrospectively to measure organ system loss of function or physiological decompensation. Coded using the All Patient Refined Diagnosis Related Groups (APR-DRGs) [12].	Integral:1 = mild, 2 = moderate, 3 = major, 4 = severe.
Comorbid conditions	Clinical conditions that exist before admission are not related to the principal reason for hospitalization and are likely to be significant factors influencing mortality and resource use [13].	Each of 30 comorbid medical conditions is treated as a dichotomous variable:0 = no, the condition was not present at time of admission,1 = yes, the condition was present at the time of admission.
Past hospitalizations during the study period	The number of previous hospitalizations that the patient experienced during the study period.	Integral: 0 = no previous hospitalizations, 1 = 1 previous hospitalization, 2 = 2 previous hospitalizations, 3 = 3 previous hospitalizations, and 4 = 4 or greater previous hospitalizations.

Context of care variables		
Average CGPR-RN	For an entire visit, the average number of all hourly CGPR RN values [14] for the visit. The hourly CGPR RN values serve as the building blocks for this variable and are calculated by dividing the total RN hours for a one-hour period by the total patient hours for that same 1-hour time period.	For each 1 hour of the visit, calculate:total no. of RN hours for a 1-hour time periodtotal no. of patient hours for that same hourand then calculate:sum of hourly CGPR RN values for the entire hospitalizationtotal hours of hospitalization.
Nursing skill mix	Proportion of RNs to all nursing direct caregivers for a specified time period.	The average of the hourly RN values was obtained by dividing the total number of RNs for all hours by the total number of hours for the hospital visit. The average of the total caregiver hours was obtained by dividing the total number of caregivers for all hours by the total number of hours for the hospital visit.
CGPR RN dip variable	The extent to which the minimum amount of RN care falls below the average of all the hourly CGPR values for the entire visit. This represents the variability in the amount of RN care that is available, specifically the extent to which the amount of RN care available drops below the average amount of RN care available for the hospital visit.	Average CGPR-RN minus the average of the three lowest hourly CGPR RN values for the visit. The larger this value is, the more the minimum CGPR RN fell below the average for the visit.
Number of units resided on	The sum of the number of units on which treatment was provided to an individual patient during the course of the hospital visit.	Integral:1 = 1 unit, 2 = 2 units, 3 = 3 units, 4 = 4 units, 5 = ≥ 5 units.

Treatments		
Number of medical treatments	Medical procedures performed during a hospital visit to diagnose and treat a given patient based upon a physician's judgment and knowledge to promote or maintain health, cure diseases, or palliate incurable diseases. Coded using ICD-9-CM codes [10] from the medical record abstraction (MRA) and regrouped into multilevel CCS categories [11].	Continuous: a count on the number of medical treatments that were performed during the course of a hospital visit, this is not the number of unique medical treatments.
Types of medical treatments	Any procedure that, based upon a physician's judgment and knowledge, is necessary to promote or maintain health, cure diseases, or palliate disease processes that are incurable. Coded using ICD-9-CM codes [10] from the MRA and regrouped into multilevel CCS categories [11].	Dichotomous:0 = no the treatment (i.e., as represented by a particular CCS category) was not received during hospitalization, 1 = yes, the treatment (i.e., as represented by a particular CCS category) was received at least once during hospitalization.
Number of unique medications	The count per visit of unique generic drug names for drugs administered at least once during a visit. Medication types were coded using the American Hospital Formulary Service's (AHFS) three-level system [9].	Continuous:a count of the number of unique medications delivered during a hospital visit.
Pharmacy treatments	Medications used in the care of patients during a hospital visit. Medication types were coded using the AHFS three-level system [9].	Dichotomous:0 = no medication from the AHFS class was administered during the hospital visit,1 = yes, at least one medication from the AHFS class was administered at least once during the hospital visit.
Number of unique nursing treatments	The number of unique nursing treatments delivered during the hospital visit. Captured using NIC [8, 15].	Continuous:a count of the unique nursing treatments delivered during the hospital visit.
Nursing treatments	Any treatment nursing personnel performed to enhance patient outcomes. Captured using NIC [8, 15].	Categorical: (multilevel) [16].(a) NIC used in >95% of visits; divided into quartiles 1 = 1–25% (lowest use rates, includes 0 use), 2 = 26–50% quartile, 3 = 51–75% quartile, 4 = 76–100% quartile (highest use rates).(b) NIC used in ≤95% and >5% of visits; divided into thirds: 0 = NIC not used, 1 = 1–33% lowest third, 2 = 34–67% middle third, 3 = 68–100% top third.(c) NIC used in <5% of the visits 0 = did not receive the NIC, 1 = did receive the NIC.
		

**Table 2:** Results from the model building process for determining explanatory variables of experiencing an adverse incident.

Variable	Significant *P* values (*P* ≤ 0.15) for within block correlations	Significant *P* values (*P* ≤ 0.05) for the final model
Patient characteristics		
Ethnicity	0.0029	
Site admitted from	<0.0001	

Clinical conditions		
Primary medical diagnoses (% of sample)		
Cancer, other primary (1.7)	<0.0001	0.0010
Maintenance chemotherapy, radiotherapy (1.1)	0.1408	
Fluid and electrolyte disorder (1.6)	0.0172	
Senility & organic mental disorders (3.0)	<0.0001	0.0140
Affective (2.1)	0.0007	
Other nervous system disorders (1.1)	0.0686	0.0176
Respiratory (3.1)	0.0687	
Chronic obstructive pulmonary (1.8)	0.0332	
Symptoms, signs, and ill-defined conditions (1.8)	0.1034	
Severity of illness	<0.0001	
Congestive heart failure (11.8)	0.0155	
Other neurological disorders (3.6)	0.1218	
Diabetes (17.7)	0.0347	
Peptic ulcer disease without bleeding (4.4)	0.0985	
Rheumatoid arthritis/collagen vas (4.0)	0.0918	
Psychoses (5.7)	0.0211	
Depression (6.6)	0.0237	

Severity of illness		
Severity of illness	<0.0001	

Elixhauser comorbid conditions (% of sample)		
Congestive heart failure (11.8)	0.0155	
Other neurological disorders (3.6)	0.1218	
Diabetes (17.7)	0.0347	
Peptic ulcer disease without bleeding (4.4)	0.0985	
Rheumatoid arthritis/collagen vas (4.0)	0.0918	
Psychoses (5.7)	0.0211	
Depression (6.6)	0.0237	

Past hospitalizations		
Past hospitalizations	0.0199	

Context of care variables		
Number of units resided on	<0.0001	
CGPR dip proportion	<0.0001	0.0092
Skill mix	0.0003	
Average caregiver patient ratio	<0.0001	<0.0001

Treatments		
Medical treatments		
Total number of procedures	<0.0001	0.0059
Types of medical treatments (% of sample)		
Incision and excision of CNS (2.0)	0.0059	
Incision of pleura, thoracentesis, chest drainage (3.8)	0.0637	
Coronary artery bypass graft (CABG) (3.1)	<0.0001	
Diagnostic cardiac catheterization, coronary arteriography (7.9)	0.0007	
Other therapeutic procedures, hemic and lymphatic system (2.8)	0.1205	
Upper gastrointestinal endoscopy, biopsy (6.6)	0.0062	
Gastrostomy, temporary and permanent (1.5)	0.1035	
Oophorectomy, unilateral & bilateral (1.3)	0.0062	
Partial excision bone (1.5)	0.0769	
Treatment of fracture or dislocation (2.3)	0.0513	
Arthroplasty (3.0)	0.0014	
Amputation of lower extremity (1.1)	0.1257	
Spinal fusion (1.0)	0.0089	
Debridement of wound, infection or burn (1.5)	0.0395	
Arterio or venogram (not heart or head) (2.2)	0.0091	
Diagnostic ultrasound (33.5)	0.0048	
Radioisotope scan (6.6)	0.0667	
Physical therapy (4.7)	<0.0001	0.0015
Psychological and psychiatric evaluation and therapy (1.8)	<0.0001	
Enteral and parenteral nutrition (9.5)	0.0063	

Pharmaceutical treatments		
Number of unique medications	<0.0001	<0.0001

Types of pharmaceutical treatments (% of sample)		
Sympathomimetic (adrenergic) agents (17.3)	0.0241	
Anticholinergic agents (13.5)	0.0054	
Skeletal muscle relaxants (5.4)	0.0140	
Cardiac drugs (64.8)	0.0445	
Hypotensive agents (37.7)	0.0882	
Psychotherapeutic agents (35.0)	<0.0001	
Succinimides (27.8)	<0.0001	0.0015
Miscellaneous central nervous system agents (3.9)	0.0923	
Opiate antagonists (1.4)	0.0669	
Anorexigenic agents and respiratory & cerebral stimulants (1.4)	0.0146	
Caloric agents (51.8)	0.0244	0.0128
Irrigating solutions (7.3)	0.0414	
Ammonia detoxicants (2.7)	0.0785	0.0274
EENT anti-infectives (42.2)	0.0002	0.0148
EENT carbonic anhydrase inhibitors (2.2)	0.0404	
Miscellaneous GI drugs (59.8)	0.1098	
Parathyroid (1.4)	0.0228	
Anti-infectives (21.5)	0.0346	
Anti-inflammatory agents (6.8)	0.0438	
Multivitamin preparations (18.7)	0.0425	
Vitamin B complex (7.4)	0.1130	
Unclassified therapeutic agents (34.0)	0.0619	
Tetracyclines (1.3)	0.1135	
Opiate agonists (64.0)	0.0034	
Barbiturates (2.8)	0.0014	
Benzodiazepines (56.2)	0.0024	
Misc. anxiolytics, sedatives, & hypnotics (17.8)	0.0022	

Nursing treatments		
Nursing treatment types (% of sample)		
Fluid management (99.5)	0.0098	
Bathing (93.5)	0.0600	
Pressure ulcer care (91.5)	<0.0001	0.0005
Bowel management (88.2)	0.1049	
Teaching (81.5)	0.0003	
Discharge planning (76.0)	0.0042	
Routine care: adult (56.2)	0.0626	
Health screening (48.8)	<0.0001	<0.0001
Sleep enhancement (47.7)	0.0572	
Oxygen therapy (42.4)	0.0008	
Post-op care (27.8)	<0.0001	
Wound care (21.4)	0.0137	
Neurologic monitoring (20.2)	0.0002	0.0003
Analgesic administration (17.2)	0.0723	
Fluid/electrolyte monitoring (15.1)	0.0365	
Medication management (12.2)	0.0678	
Nutrition management (11.3)	0.0022	
Embolus precautions (9.4)	0.0687	
Infection protection (8.9)	0.0182	
Enteral tube feeding (9.4)	0.0042	
Blood products administration (8.6)	0.0004	0.0192
Restraint (8.5)	<0.0001	<0.0001
Postprocedure care (5.6)	0.0219	
Specimen management (5.3)	0.0079	0.0098
Active listening (4.8)	0.0161	0.0003
Surgical preparation (4.1)	0.1281	0.0441
Total parenteral nutrition (TPN) administration: adult (3.4)	0.0033	
Aspiration precautions (3.2)	0.0233	
Anger control assistance (2.8)	0.0177	
Mood management (2.5)	0.0091	0.0004
Self-care assistance (2.2)	0.1323	
Procedure preparation (2.1)	0.1079	
Dementia management (1.6)	0.0816	
Electroconvulsive therapy (1.6)	0.0290	
Cast care: maintenance (1.1)	0.0035	0.0037
Splinting (1.1)	0.0086	
Music therapy (1.1)	0.0036	0.0019
Medical immobilization (0.9)	0.0356	

**Table 3:** Final model for the explanatory variables of experiencing an adverse incident.

Variable names	Estimate	Standard error	*P* value	Odds ratio	95% C.I.	
Patient characteristics						
Age at admission	−0.0001	0.00375	0.9735	1.000	0.993	1.007

Clinical conditions						
Primary medical diagnoses						
Other nervous system disorders	−0.8502	0.3623	0.0189	0.427	0.210	0.869
Cancer, other primary	0.6602	0.1957	0.0007	1.935	1.319	2.840
Senility and organic mental disorders	0.4532	0.1704	0.0078	1.573	1.127	2.197
Severity of illness						
Severe/extreme	0.3116	0.1530	0.0417	1.366	1.012	1.843
Major	0.2795	0.1379	0.0427	1.322	1.009	1.733
Moderate (*mild is reference category) *	0.1513	0.1355	0.2644	1.163	0.892	1.517
Past hospitalizations						
Four or more previous hospitalizations	−0.1976	0.2580	0.4437	0.821	0.495	1.361
Three previous hospitalizations	−0.0810	0.2460	0.7419	0.922	0.569	1.494
Two previous hospitalizations	−0.1733	0.1625	0.2864	0.841	0.611	1.156
One previous hospitalization (*no previous hospitalizations is reference) *	0.0133	0.0888	00.8809	1.013	0.852	1.206

Context of Care						
Average CGPR RN for Hospitalization (mean RN HPPD = 9.47) [Best Staffing]	−0.2817	0.1002	0.0049	0.755	0.620	0.918
Average CGPR RN for hospitalization (mean RN HPPD = 6.64)	−0.4737	0.1007	<0.0001	0.623	0.511	0.758
Average CGPR RN for hospitalization (mean RN HPPD = 5.56)	−0.0861	0.0917	0.3480	0.918	0.767	1.098
Average CGPR RN for hospitalization (mean RN HPPD = 4.07) [worse staffing]						
CGPR RN dip proportion	0.6771	0.2647	0.0105	1.150 (per 0.2 increments)	1.172	3.307

Treatments						
Medical treatments						
Number of medical treatments	0.0279	0.0140	0.0460	1.028	1.000	1.057
Physical therapy	0.4192	0.1307	0.0013	1.521	1.177	1.965
Pharmacy treatments						
Number of unique medications	0.0431	0.00438	<0.0001	1.044	1.035	1.053
Succinimides	0.2175	0.0707	0.0021	1.243	1.082	1.428
Caloric agents	0.2002	0.0792	0.0115	1.222	1.046	1.427
Ammonia detoxicants	−0.4192	0.1811	0.0206	0.658	0.461	0.938
EENT anti-infectives	0.1705	0.0730	0.0195	1.186	1.028	1.368
Nursing treatments						
Pressure ulcer care						
High use (68–100%) 0.92 use rate	0.00989	0.1555	0.9493	1.010	0.745	1.370
Medium use (34–67%) 0.41 use rate	0.3385	0.1390	0.0149	1.403	1.068	1.842
Low use (1–33%) 0.25 use rate	0.3472	0.1361	0.0107	1.415	1.084	1.848
Health screening						
High use (68–100%) 0.72 use rate	−0.2464	0.1366	0.0713	0.782	0.598	1.022
Medium use (34–67%) 0.21 use rate	−0.0938	0.0969	0.3332	0.910	0.753	1.101
Low use (1–33%) 0.08 use rate	0.2803	0.0776	0.0003	1.323	1.137	1.541
Neurologic monitoring						
High use (68–100%) 7.56 use rate	−0.1478	0.1462	0.3119	0.863	0.648	1.149
Medium use (34–67%) 4.46 use rate	0.0110	0.1308	0.9328	1.011	0.782	1.306
Low use (1–33%) 1.96 use rate	0.4180	0.1062	<0.0001	1.519	1.234	1.870
Blood products administration						
High use (68–100%) 3.70 use rate	−0.1061	0.1847	0.5657	0.899	0.626	1.292
Medium use (34–67%) 0.89 use rate	0.4029	0.1465	0.0060	1.496	1.123	1.994
Low use (1–33%) 0.17 use rate	0.1758	0.1498	0.2405	1.192	0.889	1.599
Restraint						
High use (68–100%) 16.47 use rate	0.7698	0.1554	<0.0001	2.159	1.592	2.928
Medium use (34–67%) 4.79 use rate	0.6229	0.1471	<0.0001	1.864	1.397	2.487
Low use (1–33%) 1.19 use rate	0.4595	0.1423	0.0012	1.583	1.198	2.092
Specimen management						
High use (68–100%) 1.68 use rate	−0.1680	0.2255	0.4564	0.845	0.543	1.315
Medium use (34–67%) 0.34 use rate	0.3912	0.1879	0.0374	1.479	1.023	2.137
Low use (1–33%) 0.10 use rate	0.4334	0.1767	0.0142	1.543	1.091	2.181
Active listening 1.79 use rate	0.4895	0.1385	0.0004	1.631	1.244	2.140
Mood management 3.09 use rate	0.6080	0.1748	0.0005	1.837	1.304	2.587
Cast care maintenance 5.31 use rate	0.6905	0.2335	0.0031	1.995	1.262	3.153
Music therapy 0.21 use rate	0.7102	0.2287	0.0019	2.034	1.300	3.185

## References

[1] Institute of Medicine (2000). *To Err Is Human: Building a Safer Health System*.

[2] U.S. Department of Health and Human Services Centers for Medicare and Medicaid Services http://www.cms.gov/HospitalAcqCond/06_Hospital-Acquired_Conditions.asp.

[3] Battles JB, Lilford RJ (2003). Organizing patient safety research to identify risks and hazards. *Quality and Safety in Health Care*.

[4] Duckers M, Faber M, Cruijsberg J, Grol R, Schoonhoven L, Wensing M (2009). Safety and risk management interventions in hospitals: a systematic review of the literature. *Medical Care Research and Review*.

[5] Titler M, Dochterman J, Xie XJ (2006). Nursing interventions and other factors associated with discharge disposition in older patients after hip fractures. *Nursing Research*.

[6] Titler M, Dochterman J, Picone DM (2005). Cost of hospital care for elderly at risk of falling. *Nursing Economics*.

[7] Titler M (2000). Nursing interventions and outcomes effectiveness in 3 older populations.

[8] Dochterman JM, Bulechek GM (2004). *Nursing Interventions Classification (NIC)*.

[9] McEvoy GK (2000). *American Hospital Forumlary Service (AHFS) Drug Information 2000*.

[10] Public Health Service and Health Care Financing Administration (1994). *ICD-9-CM: International Classification of Diseases, 9th Revision, Clinical Modification*.

[11] Agency for Healthcare Research and Quality (AHRQ) (2002). *Healthcare Cost and Utilization Project (HCUP)*.

[12] 3M Health Information Systems (1993). *All Patient Refined Diagnosis Related Groups (APR-DRGs)*.

[13] Elixhauser A, Steiner C, Harris DR, Coffey RM (1998). Comorbidity measures for use with administrative data. *Medical Care*.

[14] Budreau G, Balakrishnan R, Titler M, Hafner MJ (1999). Caregiver-patient ratio: capturing census and staffing variability. *Nursing Economics*.

[15] Titler M, Dochterman J, Reed D (2004). *Guideline for Conducting Effectiveness Research in Nursing and Other Health Services*.

[16] Reed D, Titler MG, Dochterman JM, Shever LL, Kanak M, Picone DM (2007). Measuring the dose of nursing intervention. *International Journal of Nursing Terminologies and Classifications*.

[17] Iezzoni L (2003). *Risk Adjustment for Measuring Health Care Outcomes*.

[18] Cho SH, Ketefian S, Barkauskas VH, Smith DG (2003). The effects of nurse staffing on adverse events, morbidity, mortality, and medical costs. *Nursing Research*.

[19] Dunton N, Gajewski B, Taunton RL, Moore J (2004). Nurse staffing and patient falls on acute care hospital units. *Nursing Outlook*.

[20] Hall LM, Doran D, Pink GH (2004). Nurse staffing models, nursing hours, and patient safety outcomes. *Journal of Nursing Administration*.

[21] Potter P, Barr N, McSweeney M, Sledge J (2003). Identifying nurse staffing and patient outcome relationships: a guide for change in care delivery. *Nursing Economics*.

[22] Sovie MD, Jawad AF (2001). Hospital restructuring and its impact on outcomes: nursing staff regulations are premature. *Journal of Nursing Administration*.

[23] Unruh L (2003). Licensed nurse staffing and adverse events in hospitals. *Medical Care*.

[24] Whitman GR, Kim Y, Davidson LJ, Wolf GA, Wang SL (2002). The impact of staffing on patient outcomes across specialty units. *Journal of Nursing Administration*.

[25] Woolcott JC, Richardson KJ, Wiens MO (2009). Meta-analysis of the impact of 9 medication classes on falls in elderly persons. *Archives of Internal Medicine*.

[26] Agostini JV, Baker DI, Bogardus ST, Shojania KG, Duncan BW, McDonald KM (2001). Prevention of falls in hospitalized and institutionalized older people. *Making Health Care Safer: A Critical Analysis of Patient Safety Practices. Evidence Report/Technology Assessment no. 43*.

[27] Evans D, Wood J, Lambert L (2002). A review of physical restraint minimization in the acute and residential care settings. *Journal of Advanced Nursing*.

[28] Hoff T, Jameson L, Hannan E, Flink E (2004). A review of the literature examining linkages between organizational factors, medical errors, and patient safety. *Medical Care Research and Review*.

[29] Kane RL, Shamliyan T, Mueller C, Duval S, Wilt TJ (2007). Nurse staffing and quality of patient care. *Evidence Report/Technology Assessment*.

[30] Picone DM, Titler MG, Dochterman J (2008). Predictors of medication errors among elderly hospitalized patients. *American Journal of Medical Quality*.

